# ADVANCING GEOGRAPHIES OF AGING AND THE LIFE COURSE

**DOI:** 10.1093/geroni/igae098.2121

**Published:** 2024-12-31

**Authors:** Jessica Finlay, Michael Desjardins

**Affiliations:** Chair; Co-Chair

## Abstract

Aging and related health outcomes are not solely individual-level processes. Rather, built, social, and natural environments play a pivotal role in shaping health across the life course, especially among older adults. People also shape and modify surrounding environments throughout their lives in meaningful ways to maximize their well-being. While multiple longitudinal health datasets are available, many are analyzed without linking to spatially-explicit data. Overlooking the nexus between health and place, and not directly accounting for spatial and spatiotemporal processes that underlie the data, may hinder scientific discovery and advancement in understanding multi-scalar longitudinal processes that impact health and well-being. Geographies of aging and the life course critically integrate built, social, and natural environments into meaningful analyses and insights that can inform research, policy, and programs to promote healthy aging. This symposium features talks using innovative qualitative, quantitative, and mixed methods studies to investigate multifaceted and overlapping contextual influences on health. The presenters critically consider mobility across the life course, multi-scalar social determinants of health, environmental impacts of structural racism and classism, and potential interactions among individuals and neighborhoods that influence vulnerability to health exposures. We aim to build capacity around spatial methods and place-based considerations in gerontology. The approaches featured in this symposium highlight accessible ways for scholars across a variety of disciplines to advance understanding of healthy aging through the integration of geospatial approaches.

